# The influence of dietary supplementation with oyster mushroom waste on laying hens’ performance, egg quality and immune parameters

**DOI:** 10.1016/j.psj.2024.104320

**Published:** 2024-09-11

**Authors:** Agori Karageorgou, Despoina Mouiki, Dimitra-M. Lolou, Ariadne-L. Hager-Theodorides, Georgios Theodorou, Theofilos Massouras, Panagiota Diamantopoulou, Panagiotis Simitzis, Ioannis Politis, Michael Goliomytis

**Affiliations:** ⁎ Department of Animal Science, Agricultural University of Athens, Athens 11855, Greece; † Department of Food Science & Human Nutrition, Agricultural University of Athens, 11855, Athens, Greece; ‡ Institute of Technology of Agricultural Products, Lykovryssi 14123, Greece

**Keywords:** mushroom waste, laying hen, oxidative stability, egg quality, cholesterol

## Abstract

The objective of the present study was to evaluate the effects of dietary supplementation with oyster mushroom (*Pleurotus ostreatus*) waste (**OMW**) on performance, egg quality, fatty acid (**FA**) profile and oxidative stability, serum and yolk cholesterol and immune parameters of laying hens. Two hundred fifty-six laying hens were allocated into 4 treatment groups, with eight replicate cages, and were fed for 28 d either a control diet, or diets supplemented with OMW at 1, 2 or 4 g per 100 g feed (P1, P2 and P4 experimental groups, respectively). No significant effects were detected on the performance and egg quality (*P* > 0.05) except from a modest decrease in the intensity of orange yolk color from hens fed with OMW, as indicated by the reduced DSM YolkFan score and color parameter a* (redness) (*P* < 0.05), and the tendency for increased color parameter L* (lightness) (*P* < 0.1). Serum total cholesterol and high-density lipoprotein content were decreased in OMW experimental groups compared with the control group (*P*, P-linear < 0.05) whereas yolk cholesterol content was unaffected (*P* > 0.05). The ratio of heterophils to lymphocytes were not influenced by dietary treatment (*P* > 0.05), whereas T helper lymphocyte (**Th**) percentage was increased in OMW supplemented groups in comparison with control (*P* < 0.05, *P-*quadratic < 0.05). The yolk FA profile was beneficially affected, as shown by the linear increase in polyunsaturated FA and a linear decrease in saturated FA in OMW supplemented groups of hens (*P*-linear < 0.05), with most notable effects observed in the P4 group. Furthermore, oxidative stability, that was expressed as malondialdehyde content, of both fresh and stored egg yolks for up to 90 d, was significantly improved in OMW supplemented groups (*P* < 0.05). The beneficial effects of OMW on yolk oxidative stability and FA profile, without adverse effects on performance or egg quality, shows that this byproduct may be successfully employed in laying hens’ diets, in a circular economy scheme, with benefits not only for the consumers and farmers but for the environment as well.

## INTRODUCTION

Oyster mushrooms (*Pleurotus* spp.) are the second most widely cultivated mushrooms globally, after shiitake, and ahead of mushrooms of the *Agaricus* and *Flammulina* genera, that are mostly grown in Asia, America and Europe (Royse et al., 2017). *Pleurotus* species are rich in protein, polysaccharides, fiber, minerals, and vitamins and display antitumor, hypocholesterolemic, immunomodulatory (Cohen et al., 2002) antiviral, antibacterial (Enshasy et al., 2017), anti-inflammatory (Taofiq et al., 2015) and antioxidant (Angelescu and Vamanu, 2015; Diamantopoulou et al., 2023) activities.

Apart from their beneficial and health promoting nutritional profile, mushrooms’ importance in the food chain relies on their ability to turn waste into food and therefore mushroom cultivation may successfully support a sustainable circular economy production system. However, mushroom cultivation is accompanied by production of by-products such as non-compliant fruiting bodies, mycelium, stem, and spent mushroom substrate (Antunes et al., 2020) that should be adequately treated to minimize their environmental impact. Rejected fruiting bodies and stems are considered as waste but at the same time they are nutritious, because chemical composition of the misshapen mushrooms is actually not different than normal ones (Guo et al., 2022), and stems have comparable chemical composition with fruiting bodies except from a higher fiber content (Oyetayo et al., 2007). Therefore, utilization of mushroom waste as animal feed may be a promising option with benefits for both the environment and animal production. The benefits of feeding mushroom waste to animals are not limited to their nutritional value but are also related to the bioactive compounds present in mushroom waste (Antunes et al., 2020). Moreover, the ban in the use of antibiotics, as growth promoters in animal production in the EU, has shifted research towards natural feed alternatives, especially for monogastric animals like poultry.

In the last decade, there is an increasing interest of the scientific community on assessing the effects of dietary supplementation from a variety of mushroom species byproducts on poultry production. *Flammulina velutipes* stems have been evaluated as a feed additive for their effects on performance, immune response and antioxidant capacity in broiler chickens (Mahfuz et al., 2020; Mahfuz et al., 2019b). Their effect on performance, calcium utilization, immunity traits (Mahfuz et al., 2018a) and antioxidant capacity (Chen et al., 2019) has also been studied in laying hens. *Cordyceps militaris* waster medium has been reported to improve growth performance and antioxidant capacity in broiler chickens (Hsieh et al., 2021) and decrease egg yolk cholesterol and feed conversion ratio in laying hens (Wang et al., 2015). *Pleurotus* species cultivation byproducts such as waste (Lee et al., 2012; Fard et al., 2014; Hassan et al., 2020), fermented waste (Lai et al., 2015) or spent mushroom substrate (Foluke et al., 2014; Adetunji and Adejumo, 2019; Mthana and Mthiyane, 2024) have been investigated in broiler chickens for their effects on performance, carcass traits or immune response. However, research on the effects of dietary oyster mushroom byproducts on performance, egg quality and health of laying hens is scarce.

Therefore, the aim of the present study was to evaluate the effect of dietary supplementation of laying hens with *P. ostreatus* mushroom waste on performance, egg quality, yolk fatty acid profile, serum and yolk cholesterol, immune parameters and yolk oxidative stability.

## MATERIAL AND METHODS

### Ethics Approval

The methods used in the present experiment were approved by the Research Ethics Committee of the Agricultural University of Athens under the protocol number 6/2023.

### Animals, Housing, and Experimental Design

A total of 256 thirty-five-wk-old ISA Brown laying hens were randomly allocated into 4 treatment groups, in the premises of the experimental farm of Agricultural University of Athens. Each treatment group consisted of 8 replicate enriched cages with 8 hens each. The experimental groups were as follows: the control (**C**) that was offered a commercial basal diet, the P1 group that was offered a diet supplemented with 1 % dried oyster mushroom waste (**OMW**) of the *Pleurotus ostreatus* species, and the P2 and P4 groups that were fed with diets supplemented with 2 and 4% OMW, respectively. All diets were isocaloric and isonitrogenous and in mash form. Table 1 presents the ingredients and the composition of the experimental diets whereas Table 2 presents their fatty acid (**FA**) profile. *P. ostreatus* waste was provided by a mushroom farm (Manitus S.A., Athens, Greece) and consisted of stems rejected during harvesting and processing of fruiting bodies. The experiment lasted for 28 days. Feed and water were provided *ad libitum,* and the light regimen was 16 h of continuous light per day. Feed intake and feed conversion ratio (**FCR**) were recorded weekly whereas egg production and egg mass were determined daily. At the 4th and 14th day of the experiment, 8 eggs were randomly selected from each treatment group (one egg from each cage, 32 eggs total), for egg yolk oxidative stability assessment by determination of the malondialdehyde (**MDA**) content. At the end of the experiment, 4 eggs were randomly collected from each cage (128 eggs total) for egg quality and oxidative stability assessment on fresh eggs and eggs stored for 28 days at room temperature (15–20°C) and for 90 days at 4°C. Egg yolk cholesterol level was also determined in the 32 fresh eggs collected. From a batch of 32 eggs, the yolk was separated and was stored at -20 °C for FA profile determination. At the 23rd d of the experiment all hens were vaccinated for Newcastle disease (**NDV**), LaSota strain, and the H120 strain of the infectious bronchitis disease (**IBV**) with an eye drop. At the 29th day of the experiment, blood samples from one hen per cage, 8 hens per treatment group, were collected from the brachial wing vein for cholesterol, NDV and IBV antibody titers and peripheral blood leukocyte assay.

**Table 1:** Ingredients and chemical composition of the experimental diets and oyster mushroom (*Pleurotus ostreatus)* waste (OMW) (g/100 g).

Diets	C	P1	P2	P4	OMW^1^
Ingredients					
Maize	60	56.6	59.0	57.8	
Wheat bran	5	4.4	3.9	3	
Soyabean meal 47%	23	23	23	23	
Dried *Pleurotus ostreatus* waste	0	1	2	4	
Soyabean oil	1.1	1	1.1	1.2	
Limestone	8.5	8.5	8.5	8.5	
Vitamin-trace-mineral premix^2^	2.5	2.5	2.5	2.5	
Calculated analysis					
ME, kcal/kg	2865	2862	2863	2859	
Crude protein	17.3	17.3	17.3	17.3	12.6 (N x 4.7)
Crude fiber	2.46	2.60	2.75	3.06	10.2
Ether extract	3.74	3.72	3.80	3.86	2.09
Ash	12.3	12.3	12.4	12.4	7.61
Calcium	3.92	3.92	3.92	3.91	
P (total)	0.79	0.79	0.78	0.77	
P (available)	0.48	0.48	0.48	0.48	
Lysine	0.90	0.90	0.90	0.90	
Methionine + cystine	0.75	0.75	0.75	0.75	
Sugars					40.4

1 Determined on a dry matter basis.

2 The vitamin and mineral premix provided per kg of diet: 10,000 IU of vitamin A (retinyl acetate); 3,000 IU of cholecalciferol; 30 mg of vitamin E (DL-α-tocopheryl acetate); 6 mg of menadione; 1.8 mg of thiamine; 6 mg of riboflavin; 5 mg of pyridoxin; 25 µg of cyanocobalamin; 44 mg of nicotinic acid; 13 mg of pantothenic acid; 1 mg of folic acid; 100 µg of biotin,; 10 mg of vitamin C (ascorbic acid); 500 mg of choline chloride; 1.5 mg of I; 0.15 mg of Se; 50 mg of Fe; 100 mg of Mn; 8 mg of Cu; 90 mg of Zn; 300 FTU of phytase; 5000 U of endo-1,4-β-xylanase and 4 mg canthaxanthin.

**Table 2:** The fatty acid profile of the experimental diets and oyster mushroom (*Pleurotus ostreatus)* waste (OMW).

Diets	C	P1	P2	P4	OMW
Fatty acid, g/100g fat					
C12:0 (lauric)	0.20	0.13	0.16	0.12	0.67
C14:0 (myristic)	0.13	0.12	0.16	0.14	0.59
C15:1 (ginkgolic)	0.17	0.10	0.12	0.15	0.49
C16:0 (palmitic)	14.5	13.4	13.7	13.1	14.0
C16:1n-9 (palmitelaidic)	0.15	0.12	0.14	0.16	0.92
C17:0 (heptadecanoic)	0.10	0.09	0.08	0.10	ND
C17:1 (cis-10-heptadecenoic)	ND	ND	ND	ND	0.77
C18:0 (stearic)	2.67	2.69	2.73	2.74	1.72
C18:1cis-9 (oleic)	20.7	21.5	21.6	21.1	9.92
C18:1n-7 (vaccenic)	0.97	0.95	0.96	0.96	0.95
C18:2n-6 (linoleic)	53.5	54.2	53.5	54.3	54.4
C18:3n-3 (linolenic, ALA)	3.81	3.42	3.64	3.80	0.71
C20:0 (arachidic)	0.36	0.47	0.43	0.44	ND
C22:0 (behenic)	0.30	0.31	0.36	0.37	0.45
C22:1 (erucic)	0.13	0.11	0.09	0.10	0.71
C24:0 (lignoceric)	0.24	0.26	0.27	0.16	0.60
SFA	18.5	17.5	17.9	17.2	21.1
MUFA	22.1	22.8	23.0	22.5	15.1
PUFA	57.3	57.6	57.1	58.1	55.1
n-6	53.5	54.2	53.5	54.3	54.4
n-3	3.81	3.42	3.64	3.80	0.71
n-6/n-3	14.0	15.8	14.7	14.3	76.6

C= control, P1=1 g per 100 g of feed, P2= 2g per 100 g of feed, M4= 4g per 100 g of feed.

SFA= Saturated fatty acids, MUFA= Monounsaturated fatty acids, PUFA= Polyunsaturated fatty acids, ND= Non-detectable value

### Egg Quality

Eggs collected for quality assessment were individually weighed and assessment of eggshell breaking strength (N) was applied using a Zwick Testing Machine (Model Z2.5/TN1S, Zwick GmbH and Co, Germany). The measurement was carried out on eggs set with the blunt end down with force applied at the sharp end. Afterwords, eggs were broken for Haugh units determination using a Haugh meter (Model S-8400, B.C. Ames Inc, Melrose, MA). Eggshell weight was determined, and its thickness was measured with a 0.01 mm precision thickness gauge (Peacock, Ozaki MFG. CO. Ltd, Tokyo, Japan) after removal of shell membranes. Measurements were applied at sharp, blunt end and equator, and the mean value for each egg was calculated. After the separation of albumen and yolk, the yolk weight was determined, and its color was estimated using a DSM Yolk Color Fan. Yolk color was also determined with a Miniscan XE (HunterLab, Reston, VA) chromameter set on the L* (lightness), a* (redness) and b* (yellowness) system. The chromameter was calibrated with white and black tiles.

### Blood Sampling, Serum and Yolk Cholesterol

Two blood samples were collected from the branchial vein of hens. A 0.2 mL blood sample was collected for the analysis of peripheral blood leukocytes as described below and a 2 ml sample was collected in tubes and was let to clot at room temperature for 15 min prior to centrifuge at 1,000 x *g* for 15 min. Supernatant serum was collected for cholesterol and antibody titer determination.

Serum total cholesterol and high-density lipoprotein (**HDL**) was photometrically measured at 540 nm in a spectrophotometer (Hitachi U3010 Spectrophotometer) using commercial cholesterol reagent kit (Biosis commercial kits; Athens, Greece). The method described by Pasin and Smith (1998) was applied for yolk cholesterol determination. Three g of egg yolk were diluted in 27 mL NaCl (20g/kg), stirred for 2 h, and 1 mL of this solution was further diluted with 9 mL NaCl (20 g/kg). The rest of the determination procedure was the same as described earlier for serum cholesterol.

### Flow Cytometric Peripheral Blood Leukocyte Analysis

For flow cytometric analysis of peripheral blood leukocytes (**PBL**) 200μl blood samples were collected as described above in tubes containing 5μl 0.5M EDTA. Red blood cells were lysed following a modified protocol of Yamamoto et al. (2007). Briefly, 100 μL blood were added to 900μl ammonium chloride-potassium (**ACK**) buffer (150 mMNH4Cl, 1mM KHCO3, 0.001 mM EDTA, filter-sterilize through a 0.2-μm filter). Samples were incubated on ice for 30 min and centrifuged at 300 x *g* for 5 min at 8°C. Pellets were resuspended in 1 mL ACK buffer, incubated for 15 min and centrifuged as previously. Cell pellets were washed (resuspended) in 2 mL staining buffer (PBS containing 0.01% NaAzide and 0.2% BSA) and centrifuged at 300 x *g* for 5 min at 8°C. Cells were washed again as in the previous step, resuspended in 100 μL in staining solution containing fluorophore conjugated antibodies (Abcam, Cambridge, UK), 0.25 μg mouse anti-CD44(AV6)-fluorescein isothiocyanate (**FITC**), 0.5 μg mouse anti-CD4(CT-4)-phycoerythrin (**PE**), and 4 μg mouse anti-CD3(**CT-3**)-PE/Cyanine (**Cy**)5 and incubated on ice for 30 min, in the dark. Stained cells were washed in staining buffer as described above, resuspended in 200 μL staining buffer and analyzed on a Cytomics FC 500 flow cytometer (Beckman Coulter Inc. Brea, CA).

Instrument voltage/gain for detectors FS (Linear), SS (Linear), FL1 (Log, FITC detection), FL2 (Log, PE detection), FL3 (Log) and FL4 (Log, PE-Cy5 detection) were set at 600/2.0, 695/20.0, 600/1.0, 600/1.0, 500/1.0, 620/1.0, respectively. Samples were run at maximum speed and 100,000 events were collected and data was stored as list mode files.

### NDV and IBV Antibody Titer Assay

Serum from immunized hens was screened for the presence of IgY antibodies, which are the main immunoglobulin present in avian blood, against attenuated live Infectious Bronchitis, H120 strain and Newcastle disease, La Sota strain. The total IgY titers were measured using an enzyme-linked immunosorbent assay (**ELISA**) with the following procedure. First, 10 mL PBS were added to commercial vaccine vials for Infectious Bronchitis H120 and Newcastle disease La Sota strains and gently shaken until the freeze-dried tablet was completely dissolved, following manufacturer's instructions. The solutions were further diluted in a total volume of 30ml PBS resulting in a concentration of 0,88mg/ml and 4,4mg/ml respectively. Next, 96-well High Binding plates were coated with each antigen at a concentration of 10ug/ml and left to incubate overnight at 4 °C. After blocking with 5% Milk for 2h at 37 °C, sera were diluted 1:10 and incubated in duplicates for 30 minutes at room temperature. Following PBST (PBS with 0.05% Tween) washes, mouse a-IgY Ab was added at a dilution of 1:10 from a 100 ug/mL stock. After a 30-minute incubation, goat anti-mouse IgG Ab conjugated with horseradish peroxidase was added and incubated for another 30 minutes. Subsequently, the substrate 3, 3′, 5, 5′-Tetramethylbenzidine was added for 4 min, and the reaction was terminated using Phosphate Buffer. The amount of chromogen produced was measured based on absorbance at 450 nm. Antibody positive cut-off values were set as means+2 standard deviations of PBS control group.

### Egg Yolk Oxidative Stability

Assessment of egg yolk oxidative stability was applied by determination of MDA, a secondary lipid oxidation product formed by hydrolysis of lipid hydroperoxides. Yolk MDA concentrations were determined by using a selective third-order derivative spectrophotometric method developed by Botsoglou et al. (1994). Two g of each sample (2 samples per egg) were homogenized (Edmund Buehler 7400 Tuebingen/H04, Germany) in the presence of 8 ml aqueous trichloroacetic acid (50 g/L) and 5 mL butylated hydroxytoluene in hexane (8 g/L), and the mixture was centrifuged for 3 min at 3,000 x *g*. The top hexane layer was discarded and a 2.5 mL aliquot from the bottom layer was mixed with 1.5 mL aqueous 2-thiobarbituric acid (8 g/L) to be further incubated at 70°C for 30 min. Following incubation, the mixture was cooled under tap water and submitted to third-order derivative (3D) spectrophotometry (Hitachi U3010 Spectrophotometer) in the range of 500–550 nm. The concentration of MDA (ng/g yolk) in analyzed samples was calculated on the basis of the height of the third-order derivative peak at 521.5 nm by referring to slope and intercept data of the computed least-squares fit of standard calibration curve prepared using 1,1,3,3-tetraethoxypropane, the MDA precursor.

### Fatty Acid Profile

Fatty acid profile was determined in feed and yolk samples. Feed FA profile was determined in duplicate. Total cellular lipid was extracted from the dry biomass by chloroform/methanol: 2/1 (v/v) mixture and trans-methylated according to Fakas et al. (2006). Fatty acid analysis was performed by gas chromatography (**GC**), as described by Diamantis et al. (2022).

The yolk FA composition was determined by direct methylation of lyophilized yolk samples in duplicate according to Massouras et al. (2017). Briefly, lyophilized yolk was directly methylated with 2 ml of 0.5 M sodium methylate and 2 ml of boron trifluoride in methanol (BF3). Fatty acid methyl esters were then recovered in 1 ml of hexane. The FA methyl esters (FAMES) were analyzed on a Shimadzu chromatograph (model GC-17A, Kyoto, Japan) equipped with flame ionization detector. The FAMES were separated using a SP-2560 capillary column (100 m x 0.25 mm I.D., 0.20 μm; Supelco Bellefonte, PA). The split ratio was 1:50 and helium was used as the carrier with flow rate 1.8 Ml/min. The injector and detector temperatures were 250 ^∘^C and 270 ^∘^C, respectively. The injection volume was 1 μL. The initial column temperature was 45 ^∘^C (held for 3 min), then increased at 7 ^∘^C per min to 100 ^∘^C (held for 5 min) and 5 ^∘^C min to a final temperature of 220 ^∘^C (held for 20 min). The identification of the FAME peaks was performed by comparing the retention times of the FAME standards. Supelco 37 Component FAME Mix was purchased from Sigma-Aldrich (Merck KGaA, Darmstadt, Germany). A GC Solution software (Shimadzu Corporation, Kyoto, Japan) was used for the integration of the peaks. The individual fatty acid (**FA**) content was expressed as a weight percentage (g per 100 g of total FA). The different groups of FA were determined, in feed or yolks, as follows:

Saturated fatty acids (**SFA**) = C12:0 + C14:0 + C15:0 + C16:0 + C17:0 + C18:0 + C22:0 + C24:0; Monounsaturated fatty acids (**MUFA**) = C15:1 + C17:1 + C16:1n-7 + C16:1n-9 + C17:1 + C18:1n-9 + C18:1cis9 + C18:1n-7 + C20:1n-9 + C22:1; Polyunsaturated fatty acids (PUFA) = C18:2n-6 + C18:3n-6 + C18:3n-3 + C20:2n-6 + C20:3n-9 + C20:3n-6 + C20:4n-6 + C22:5n-3 + C22:6n-3.

### Statistical Analysis

Experimental data were subjected to analysis of variance with the MIXED procedure of the SAS software (SAS Institute Inc). The fixed effect was the dietary treatment with mushroom waste. The linear and quadratic dose responses to dietary mushroom waste were tested with orthogonal polynomials with the CONTRAST procedure. MDA and egg quality data were analyzed with the MIXED procedure for repeated measures. Dietary treatment, days of storage or supplementation duration for MDA measurements of fresh eggs, and their interaction were the fixed effects, whereas days of storage or duration of supplementation was the repeated factor. For MDA and egg quality traits, dose response effects were calculated within each storage period or duration of supplementation. Multiple comparisons were applied after Bonferroni adjustment. Classification of yolk samples according to the dietary treatment was evaluated by a discriminant analysis of the FA profile. A stepwise discriminant analysis was performed in order to reveal FA that were mainly responsible for the observed classification. Statistical significance was set at *P* < 0.05. Probability values of 0.05 < *P* < 0.1 were considered as tendencies to differences. Results are presented as least squares means ± SEM.

## RESULTS

Performance traits measured, that is, laying rate, feed intake, FCR and egg mass were not affected by dietary supplementation with OMW at 1, 2 and 4 g per kg of feed (Table 3, *P* = 0.736, 0.268, 0.151 and 0.706 for laying rate, feed intake, FCR and egg mass, respectively) except from a tendency for a linear increase for FCR (*P*-linear = 0.072). Egg quality traits of fresh or stored eggs, for either 28 d at room temperature or for 90 d at refrigerated storage, are presented in Table 4. Storage time had a significant effect on most quality attributes except for those directly related to eggshell. While eggshell weight percentage increased with increasing storage (*P* = 0.01), eggshell absolute weight remained unaffected (*P* = 0.331). This change is explained by the decreased absolute and relative weights of albumen because of the evaporation of its water through the shell pores during storage. Oyster mushroom waste dietary supplementation to laying hens did not affect egg quality traits except for traits related to yolk color. Redness (a*) decreased, on either fresh or stored eggs, with the addition of mushroom waste in the diets of hens (*P* < 0.001) in a linear pattern (*P*-linear = 0.087, 0.016 and 0.027 at 0, 28 and 90 d of storage, respectively) whereas, lightness (L*) significantly increased in OMW supplemented groups when eggs were stored for 90 d in the refrigerator ( -linear= 0.005). This effect was reflected in the moderate, but significant, decrease in DSM yolk color fan scores determined throughout the experiment (*P* = 0.044), from 12.0 in the C group to 11.8, 11.5 and 11.8 in the P1, P2 and P4 groups respectively, with a significant difference detected between the C and the P2 groups (*P* = 0.032). A linear dose response was detected at 28 d of storage (*P*-linear = 0.036). Nevertheless, significant treatment effects determined for egg yolk color traits were not influenced by egg storage, as shown by the lack of a significant treatment by time interaction (*P* = 0.597, 0.588, 0.345 and 0.429 for L*, a*, b* and DSM color parameters, respectively).

**Table 3:** Effect of dietary oyster mushroom (*Pleurotus ostreatus*) waste on laying hens’ performance (n=8 replicate cages).

	Treatment					*P*-values		
Variable	C	P1	P2	P4	SEM	Treatment	Linear	Quadratic
Laying rate (%)	94.2	93.1	94.8	91.4	2.30	0.736	0.432	0.622
Feed intake (g/day)	117	121	118	123	2.51	0.268	0.146	0.881
FCR (g egg/g feed)	1.91	2.02	1.93	2.10	0.06	0.151	0.072	0.664
Egg weight (g)	61.5	60.0	61.0	58.9	1.59	0.685	0.316	0.877
Egg mass (g)	58.1	56.0	57.9	54.2	2.64	0.706	0.361	0.785

C= control, P1=1 g per 100 g of feed, P2= 2g per 100 g of feed, M4= 4g per 100 g of feed.

**Table 4:** Effect of dietary supplementation with oyster mushroom (*Pleurotus ostreatus*) waste on quality of laying hens’ fresh and stored eggs (n = 8).

	Treatment						*P*-values				
Variable	Storage time, days	C	P1	P2	P4	SEM	Treatment	Time	Treatment × Time	Linear	Quadratic
Egg weight, g	0	64.4	66.8	66.2	62.9	1.69	0.132	<0.001	0.643	0.349	0.146
	28	59.5	59.4	62.1	60.5	1.59				0.516	0.452
	90	62.2	62.6	64.2	59.4	1.61				0.205	0.127
Yolk weight, g	0	16.1	16.5	16.3	14.7	0.69	0.137	0.003	0.444	0.097	0.231
	28	17.7	17.3	17.6	17.2	0.88				0.729	0.970
	90	16.5	18.6	18.3	16.8	0.55				0.879	0.002
Yolk, %	0	24.9	24.7	24.6	23.3	0.89	0.507	<0.001	0.384	0.183	0.668
	28	29.9	29.3	28.3	28.4	1.54				0.493	0.670
	90	26.5	29.8	28.5	28.4	0.71				0.290	0.026
Albumen weight, g	0	40.3	42.4	41.5	40.8	1.27	0.451	<0.001	0.687	0.969	0.320
	28	34.1	35.0	36.7	35.5	1.56				0.526	0.373
	90	37.8	35.7	37.8	34.9	1.27				0.193	0.707
Albumen weight, %	0	62.5	63.4	62.6	64.8	0.93	0.584	<0.001	0.305	0.113	0.545
	28	57.5	58.2	59.1	58.5	1.65				0.588	0.527
	90	60.6	56.9	58.7	58.7	0.87				0.447	0.068
Eggshell weight, g	0	8.08	7.9	8.42	7.49	0.25	0.350	0.331	0.387	0.142	0.118
	28	7.69	7.53	7.86	7.92	0.34				0.491	0.910
	90	8.03	8.31	8.13	7.69	0.21				0.139	0.175
Eggshell weight, %	0	12.5	11.8	12.8	11.9	0.26	0.945	0.011	0.258	0.274	0.498
	28	13.0	12.7	12.6	13.1	0.46				0.777	0.453
	90	13.4	13.4	12.7	13.0	0.37				0.797	0.955
Eggshell strength, N	0	41.3	37.8	40.0	36.2	3.10	0.203	0.021	0.890	0.323	0.981
	28	41.7	33.1	35.5	33.9	3.21				0.196	0.270
	90	34.6	32.5	30.8	32.2	3.22				0.630	0.513
Eggshell thickness, mm	0	0.43	0.40	0.41	0.40	0.01	0.421	0.777	0.286	0.190	0.993
	28	0.40	0.39	0.40	0.41	0.01				0.338	0.529
	90	0.40	0.40	0.41	0.40	0.01				0.865	0.551
Yolk colour, L*	0	58.1	61.1	58.2	58.9	1.31	0.068	0.058	0.597	0.922	0.579
	28	56.4	58.5	59.6	59.3	1.25				0.129	0.232
	90	55.8	57.4	58.1	58.3	0.54				0.005	0.088
Yolk colour, a*	0	30.7	28.8	27.6	28.8	0.70	0.015	<0.001	0.588	0.087	0.015
	28	32.8	31.2	29.0	28.2	1.32				0.016	0.427
	90	32.7	31.5	30.5	30.1	0.79				0.027	0.336
Yolk colour, b*	0	59.0	58.4	58.4	57.8	1.59	0.302	<0.001	0.345	0.589	0.937
	28	71.8	77.5	69.8	65.5	3.70				0.092	0.409
	90	68.8	70.2	71.5	69.5	1.46				0.767	0.212
Yolk color, DSM YolkFan^TM^	0	12.1	11.9	11.8	11.9	0.19	0.044	0.079	0.429	0.412	0.254
	28	12.1	11.6	11.3	11.5	0.19				0.036	0.020
	90	11.9	11.8	11.6	11.9	0.15				0.950	0.194
Haugh units	0	90.8	90.8	89.0	90.2	2.13	0.218	<0.001	0.688	0.794	0.672
	28	37.4	26.4	25.7	20.8	5.98				0.082	0.047
	90	71.0	68.2	68.4	68.4	2.39				0.529	0.340

C= control, P1=1 g per 100 g of feed, P2= 2g per 100 g of feed, M4= 4g per 100 g of feed.

L*=lightness, a*=redness, b*=yellowness.

As shown in Table 5, dietary supplementation with OMW resulted in a decrease of the hens’ serum total cholesterol and HDL content (*P* = 0.015) in a dose-dependent manner (*P*-linear = 0.010). A decrease by almost 30% was observed for total cholesterol content with significant differences being detected (*P* < 0.018) between the C (90.7 mg/dL) and P4 groups (53.6 mg/dL). Similarly, significant differences for HDL content were detected between the control (8.35 mg/dL) and the P4 groups (4.93 mg/dL). On the other hand, yolk cholesterol levels, were not affected by OMW supplementation (*P* = 0.887).

**Table 5:** Effect of dietary oyster mushroom (*Pleurotus ostreatus*) waste for 28 days on laying hens’ egg and blood serum cholesterol levels (n = 8).

	Treatment					*P*-values		
Variable	C	P1	P2	P4	SEM	Treatment	Linear	Quadratic
Blood cholesterol (mg/dl)	90.7^a^	60.4^ab^	68.3^ab^	53.6^b^	7.75	0.015	0.010	0.250
Blood HDL (mg/dl)	8.35^a^	6.39^ab^	7.08^ab^	4.93^b^	0.75	0.043	0.012	0.921
Yolk cholesterol (mg/g)	16.1	16.7	16.0	16.8	0.84	0.887	0.674	0.884
Yolk cholesterol per egg (mg)	257.6	273.2	260.4	245.5	16.4	0.719	0.456	0.485

C= control, P1=1 g per 100 g of feed, P2= 2g per 100 g of feed, M4= 4g per 100 g of feed.

a,b Means in a row sharing no common superscript are statistically different (*P* < 0.05).

**Figure 1:**
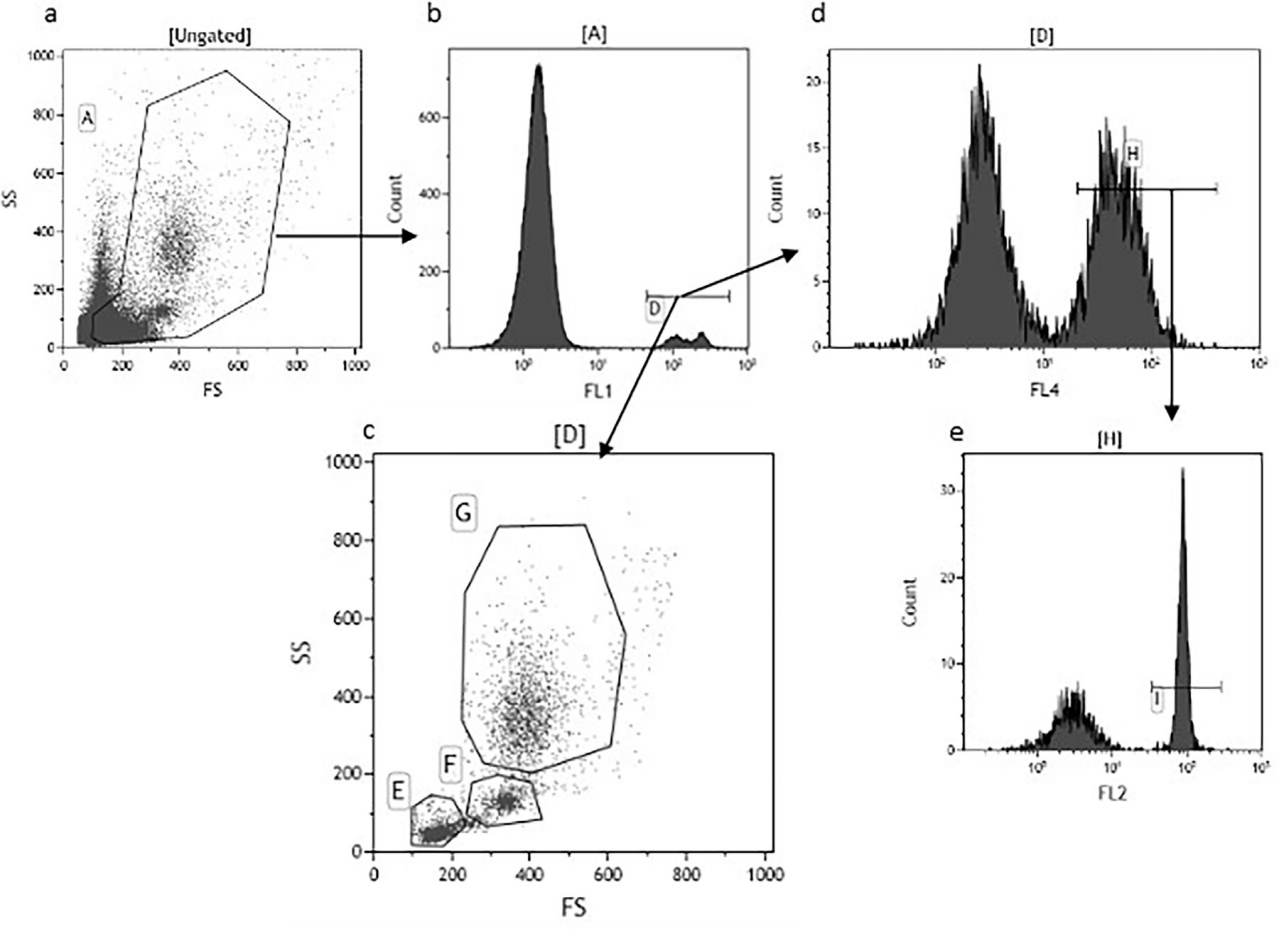
Identification of different peripheral blood leukocyte types by flow cytometry. (A) Forward/side scatter (FS/SS) dot plot of all events. (B). Events in region A are plotted on a histogram for CD44 cell surface expression and positively stained cells (region D) are identified as leukocytes. (C). Cells identified as leukocytes are plotted on an FS/SS dot plot and regions E, F and G are used to identify lymphocytes, monocytes and polymorphonuclear granulocytes respectively. The majority of polymorphonuclear granulocytes in the blood are heterophils. (D). Leukocytes are also plotted on a histogram of CD3 cell surface expression and positively stained cells are identified as T cells. (E). T cells are further plotted on a histogram of CD4 cell surface expression and positively stained cells are identified as T helper (**Th**) cells.

The percentages of different types of PBL, monocytes, heterophils, lymphocytes, T cells and T helper (**Th**) cells were determined by flow cytometry based on cell surface expression of CD44, CD3 and CD4 (Figure 1). No differences were observed in any leukocyte types (*P* > 0.05), except for Th cells (Table 6). Th cell percentage significantly increased in mushroom waste supplemented groups in comparison with control (*P* = 0.021) and a quadratic dose response was detected (*P*-quadratic = 0.011). IgY antibodies against IBV and NDV were not affected by OMW dietary supplementation of laying hens (Table 6; *P* = 0.443 and 0.910 for IBV and NDV titers, respectively).

**Table 6:** Effect of dietary oyster mushroom (*Pleurotus ostreatus*) waste for 28 days on laying hens’ immune parameters (n = 8).

	Treatment					*P*-values		
Variable	C	P1	P2	P4	SEM	Treatment	Linear	Quadratic
IBV titer	1.11	0.89	0.97	0.96	0.09	0.443	0.489	0.308
NDV titer	0.86	0.88	0.93	0.84	0.09	0.910	0.846	0.527
% Lymphocytes	39.5	42.1	44.2	45.9	3.48	0.622	0.218	0.708
% Monocytes	12.8	10.4	9.30	9.42	1.69	0.461	0.219	0.341
% Heterophils	37.7	39.9	37.7	36.6	3.52	0.932	0.709	0.750
% T cells	34.3	37.5	38.0	38.1	3.36	0.831	0.497	0.580
Th, % of T cells	33.9^a^	48.3^b^	54.4^b^	47.5^b^	4.39	0.021	0.080	0.011
H/L ratio	0.99	1.12	0.95	0.82	0.19	0.761	0.422	0.655

C= control, P1=1 g per 100 g of feed, P2= 2g per 100 g of feed, M4= 4g per 100 g of feed.

IBV=Infectious bronchitis virus Elisa antibody titer.

NDV=Newcastle disease virus Elisa antibody titer.

Th=T helper, H=Heterophils, L=Lymphocytes.

a,b Means in a row sharing no common superscript are statistically different (*P* < 0.05).

Results of repeated measures analysis on egg yolk oxidative stability throughout the experiment and during egg storage are presented in Table 7. Reduced yolk MDA values were observed for OMW fed experimental groups; overall means were 5.7, 5.2, and 5.1 ng MDA per g yolk for P1, P2 and P4 groups, respectively, compared to 6.6 ng MDA per g yolk in the control group (*P* = 0.004). This effect was evident even from the 4th d of supplementation (*P-*linear = 0.023). This improvement in yolk oxidative stability followed a linear dose response pattern also at 14th and 28th d after mushroom waste supplementation (*P*-linear=0.035 and 0.043, respectively). Supplementation duration positively affected the yolk oxidative stability response to dietary mushroom waste, as shown from the decreased average MDA values over time (6.4, 6.1 and 4.5 ng MDA per g of yolk at 4, 14 and 28 d, respectively, *P* < 0.001). No significant treatment by time interaction was detected (*P* = 0.798). Improved yolk oxidative stability in the OMW experimental groups was also observed when eggs were stored at room temperature for 30 d or in the refrigerator for 90 d (*P* = 0.017). MDA values tended to decrease in a dose dependent pattern, from 9.6 in the C group, to 8.2 ng MDA/g yolk in the P4 group at room temperature (*P*-linear = 0.085), and from 7.1 (C group) to 5.3 ng MDA/g yolk (P4 group) in refrigerated storage (*P*-linear = 0.073). Reduced MDA values were determined at refrigerated storage (5.9 ng MDA/g yolk) in comparison with storage at room temperature (8.7 ng MDA/g yolk, *P* < 0.001). No significant interaction of dietary intervention by storage time was determined (*P* = 0.978).

**Table 7:** Effect of dietary supplementation with oyster mushroom (*Pleurotus ostreatus*) waste on oxidative stability of laying hens’ fresh and stored eggs (ng of malondialdehyde/g of yolk, n = 8).

	Treatment						*P*-values			
Duration of supplementation, days	C	P1	P2	P4	SEM	Treatment	Time	Treatment x Time	Linear	Quadratic
4	7.36	6.82	5.65	5.68	0.54				0.023	0.275
14	7.33	5.88	5.63	5.58	0.50	0.004	<0.001	0.798	0.035	0.098
28	5.20	4.26	4.19	4.16	0.30				0.043	0.091
Storage time, days*										
30	9.58	8.82	8.26	8.20	0.54	0.017	<0.001	0.978	0.085	0.305
90	7.07	5.75	5.50	5.26	0.63				0.073	0.271

C= control, P1=1 g per 100 g of feed, P2= 2g per 100 g of feed, M4= 4g per 100 g of feed.

⁎ Eggs were stored for 30 days at room temperature (20° C) and for 90 days at 4° C.

**Figure 2:**
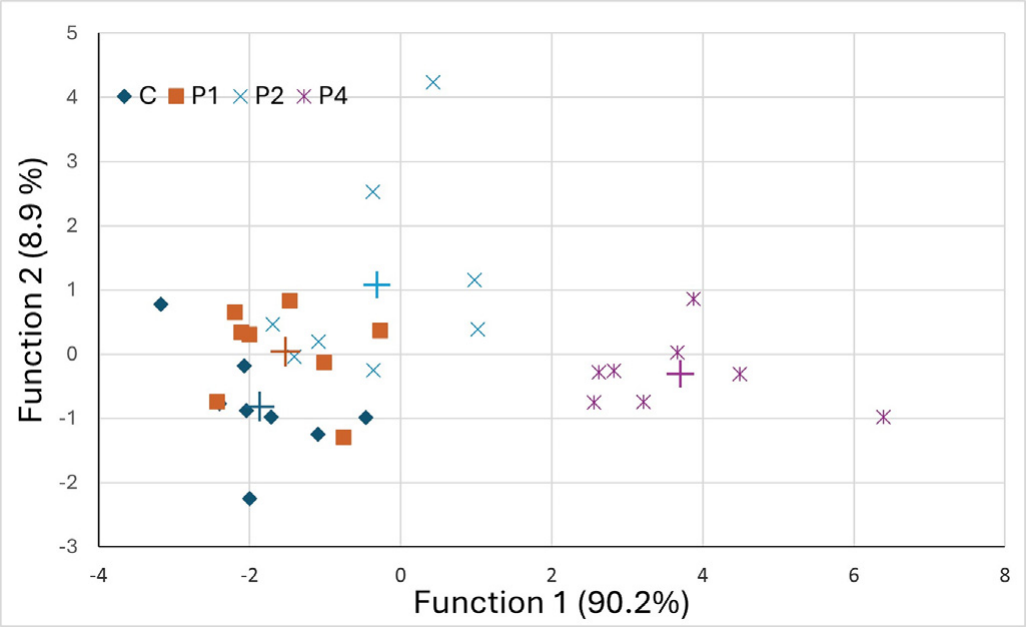
Discriminant analysis for different levels of dietary supplementation with oyster mushroom (*Pleurotus ostreatus)* waste on laying hens using 2 discriminant functions of fatty acids determined on egg yolks (+ indicates group centroid). C= control, P1=1 g per 100 g of feed, P2= 2g per 100 g of feed, M4= 4g per 100 g of feed.

The FA profile of egg yolks is presented in Table 8. Egg yolk saturated FAs (**SFA**) and monounsaturated FAs (**MUFA**) were linearly decreased, whereas polyunsaturated FAs (**PUFA**) increased linearly with increasing levels of mushroom waste in the diets of laying hens (*P*-linear < 0.001 for SFA and PUFA and *P* = 0.014 for MUFA). The most abundant FAs, that is, the saturated C16:0 and C18:0 and the monounsaturated C18:1n-9 were also decreased in a linear dose response pattern (*P*-linear=0.002, 0.033 and 0.036, respectively). The C18:1n-9 was the FA with the highest concentration and decreased by 2.2%, from 41.3 in the C group to 39.1% in the P4 group. The most abundant polyunsaturated FA was C18:2n-6 and it was linearly increased with increasing levels of dietary OMW, from 15.5% in the C group to 19.2% in the P4 group (*P*-linear<0.001). The observed PUFAs increase was attributed to the n-6 FA increase (*P* < 0.001, *P*-linear < 0.001) since n-3 FA content was not affected by dietary intervention (*P* = 0.647). In most of the significant effects detected (*P* < 0.05) in the FA profile, significant differences were observed between the P4 group and the remaining experimental groups. This was the case for C16:0, C17:0, C16:1n9, C16:1n7, C17:1, C18:2n-6, C20:1n9, C20:2n6, C20:3n9, and sum of n-6, SFAs and PUFAs. This notable effect, of the highest dietary inclusion rate of 4 g of OMW per kg of feed, on FA profile is clearly visualized in the graph of the first 2, out of 3, significant canonical functions of the discriminant analysis of FA profile (Figure 2). Yolk samples from the P4 group were clearly distinguished in a separate cluster from the yolk samples of the C, P1 and P2 groups, that formed another cluster. Stepwise discriminant analysis on classification according to diet type showed that 5 out of 22 fatty acids were responsible for the discrimination of yolk samples into dietary treatment groups. These fatty acids were C17:0, C17:1, C18:2n-6, C18:3n-3 and C20:1n-9.

**Table 8:** Effect of dietary supplementation with oyster mushroom (*Pleurotus ostreatus*) waste on fatty acid profile of laying hens’ egg-yolks (n = 8).

	Treatment					*P*-values		
Fatty acid, g/100g fat	C	P1	P2	P4	SEM	Treatment	Linear	Quadratic
C14:0 (myristic)	0.29^ab^	0.31^a^	0.30^ab^	0.26^b^	0.01	0.013	0.010	0.032
C15:0 (pentadecanoic)	0.57	0.60	0.60	0.59	0.09	0.996	0.923	0.841
C16:0 (palmitic)	26.3^a^	26.5^a^	25.9^ab^	25.1^b^	0.29	0.009	0.002	0.323
C16:1n-7 (palmitoleic)	2.68^a^	2.78^a^	2.65^a^	2.11^b^	0.10	<0.001	<0.001	0.020
C16:1n-9 (elaidic)	0.47^a^	0.49^a^	0.56^ab^	0.68^b^	0.03	<0.001	<0.001	0.453
C17:0 (heptadecanoic)	0.16^a^	0.17^a^	0.19^b^	0.25^b^	0.01	<0.001	<0.001	0.135
C17:1 (cis-10-heptadecanoic)	0.072^a^	0.074^a^	0.086^b^	0.089^b^	0.003	0.002	<0.001	0.338
C18:0 (stearic)	8.89	9.02	8.75	8.45	0.17	0.126	0.033	0.450
C18:1n-9 (oleic)	41.3	40.4	40.9	39.1	0.65	0.124	0.036	0.605
C18:2n-6 (linoleic)	15.5^a^	15.8^a^	16.1^a^	19.2^b^	0.53	<0.001	<0.001	0.076
C18:3n-6 (γ-linolenic)	0.025	0.025	0.024	0.020	0.002	0.177	0.040	0.444
C18:3n-3 (α-linolenic, ALA)	0.658	0.707	0.712	0.787	0.041	0.196	0.037	0.976
C20:1n-9 (gondoic)	0.086^a^	0.098^ab^	0.106^ab^	0.122^b^	0.008	0.034	0.004	0.806
C20:2n-6 (eicosadienoic)	0.162^a^	0.163^a^	0.159^a^	0.200^b^	0.007	<0.001	<0.001	0.022
C20:3n-9 (mead)	0.036^a^	0.037^a^	0.033^ab^	0.026^b^	0.002	0.002	<0.001	0.250
C20:3n-6 (eicosatrienoic)	0.131	0.140	0.129	0.151	0.006	0.070	0.046	0.315
C20:4n-6 (arachidonic)	1.75	1.74	1.78	1.89	0.05	0.117	0.025	0.410
C22:0 (behenic)	0.057	0.052	0.051	0.042	0.004	0.086	0.013	0.923
C22:5n-3 (eicosapentaenoic)	0.171	0.159	0.186	0.209	0.013	0.059	0.016	0.468
C22:6n-3 (docosahexaenoic)	0.474	0.492	0.469	0.460	0.062	0.985	0.794	0.892
SFA	36.3^a^	36.7^a^	35.8^ab^	34.7^b^	0.30	<0.001	<0.001	0.132
MUFA	44.6	43.8	44.3	42.1	0.65	0.054	0.014	0.390
PUFA	18.9^a^	19.3^a^	19.7^a^	23.0^b^	0.58	<0.001	<0.001	0.087
n-3	1.30	1.36	1.37	1.46	0.09	0.647	0.214	0.980
n-6	17.5^a^	17.9^a^	18.2^a^	21.5^b^	0.55	<0.001	<0.001	0.068
n-6/n-3	13.8	13.2	13.9	15.1	0.92	0.517	0.483	0.796

C= control, P1=1 g per 100 g of feed, P2= 2g per 100 g of feed, M4= 4g per 100 g of feed.

SFA=Saturated fatty acids, MUFA= Monounsaturated fatty acids, PUFA= Polyunsaturated fatty acids.

a,b Means in a row sharing no common superscript are statistically different (*P* < 0.05).

## DISCUSSION

The aim of the present study was to evaluate the effects of dietary *P. ostreatus* cultivation waste on hen performance, egg quality, blood and egg cholesterol content, immune parameters, yolk FA profile and yolk oxidative stability. Results of our study showed that dietary OMW improved yolk oxidative stability of either fresh or stored eggs in a linear dose response manner. This beneficial effect may be attributed to a variety of secondary metabolites found in mushrooms which have been shown to act as excellent antioxidants (Mau et al., 2002; Dubost et al., 2007). Oyster mushrooms contain substances that exert antioxidant activity such as tannins, coumarins, saponins and alkaloids (Fontes Vieira et al., 2013) but their phenolic compounds are the major antioxidant components that significantly contribute to their antioxidant capacity (J.-H. Yang et al., 2002) and are responsible for their *in vitro* and *in vivo* antioxidant effects (Jayakumar et al., 2011). Phenolic compounds detected in oyster mushrooms such as gallic, trans-cinnamic, caffeic, ferulic, aspartic and vanillic acid or flavonoids like quercetin (Goswami et al., 2021), neutralize free radicals by donating an H-atom or by a single electron transfer mechanism or by chelating transition metals and forming stable products (Zeb, 2020). Results of the present study showed that self-life of eggs was improved when hens were fed with OMW. This beneficial effect has also been reported by Yang et al. (2021) in either fresh or stored eggs for 21 d, after supplementing hen diets with *Agaricus bisporus* stem waste. Karaalp et al. (2018) also determined reduced MDA values, in stored eggs for 28 d in the refrigerator, in comparison to controls when they fed laying hens with *Agaricus bisporus* stalks. However, they did not observe improved oxidative stability in fresh eggs. Chen et al. (2019) in agreement with our study reported reduced yolk MDA values and consequently, improved oxidative stability in fresh eggs as a result of feeding laying hens with stem wastes from *Flammulina velutipes* mushrooms.

The observed beneficial effect of dietary *P. ostreatus* waste on yolk oxidative stability, in the present study, was not accompanied by any significant effect on performance except from a tendency for a linear FCR increase with increased levels of OMW in the diet of hens. The higher crude fiber content of the P4 diet, by 20% in comparison with the controls, may possibly explain the observed tendency because increased fiber content may negatively affect utilization of nutrients (Jha and Mishra, 2021). Egg quality was not affected by OMW supplementation except from a slight decrease in DSM yolk color values. Yolk color showed increased lightness and decreased redness during storage for 28 d with increasing levels of dried OMW in the diets of hens. In agreement with our study, Na et al. (2005) reported a less intense yolk color when hens were fed with *F. velutipes* media. On the other hand, Mahfuz et al. (2018b) reported that yolk color was improved by feeding hens with *F. velutipes* stem waste, probably because of the pigments responsible for its color. Hwang (2012) and Lee et al. (2014) did not detect any effect of dietary shitake mushrooms or *F. velutipes* mycelium fermented with *Bacillus subtilis* and *Klebsiella sp*., respectively, on yolk color of hens. Furthermore, Lee et al. (2014), determined increased values of Haugh units, shell weight and thickness at the concentration of 4 g of fermented *F. velutipes* mycelium per kg of feed. Discrepancies among published studies may be attributed not only to different mushroom species examined, but also to different raw materials fed, such as media or fruiting bodies and stems, or processing like fermentation with bacteria.

Addition of OMW in the diet of laying hens at 1, 2, or 4% reduced the level of blood total cholesterol and HDL by almost 30%, whereas egg yolk cholesterol was not influenced by dietary treatment. In agreement with our study, Mahfuz et al. (2019a) and Shang et al. (2014) reported reduced serum total cholesterol and HDL levels in broiler chickens fed with *F. velutipes* waste or polysaccharides from a fermentation concentrate of *Hericium caput-medusae*, respectively. Reduced egg yolk cholesterol levels have been reported when laying hens were fed with shiitake mushrooms (Hwang, 2012) or *Cordyceps militaris* waster medium (Wang et al., 2015). The lowering cholesterol effects of dietary mushroom may be attributed to their high dietary fibre (Brown et al., 1999) or polysaccharides (Shang et al., 2014) content. Dietary polysaccharides increase the fecal bile acids output (Shang et al., 2014) and increased bile acid excretion results in a decrease in serum cholesterol levels because cholesterol is consumed for new bile-salt synthesis (Jeun et al., 2010; Viveros et al., 2007). Another bioactive compound present in mushrooms that is responsible for their cholesterol lowering effect is mevinolin (lovastatin). Mevinolin present in *P. ostreatus* mushrooms inhibits the microsomal enzyme 3-hydroxy-3-methylglutarylcoenzymeA (HMG-CoA) reductase, which is the major rate-limiting enzyme in cholesterol biosynthesis as it converts HMG-CoA to mevalonate in the rate-limiting step in the synthesis of cholesterol (Gunde‐Cimerman et al., 1993). Consequently, inhibition of HMG-CoA reductase decreases intracellular cholesterol biosynthesis (Steinberg et al., 1989).

There are several reports indicating that mushrooms exert immunomodulatory properties (Meng et al., 2016; Bederska-Łojewska et al., 2017) and thus the effect of oyster mushroom residues on immune parameters was assessed in the present study. Previous studies have shown that dietary supplementation with mushroom residues or extracts increased antibody response to vaccination for Newcastle disease, Infectious bronchitis, Avian Influenza, sheep red blood cell, and parasitic infection in broilers (Guo et al., 2004; Hassan et al., 2020) and laying hens (Mahfuz et al., 2018a; Mahfuz et al., 2019b). In contrast, in the present study and in agreement with Toghyani et al. (2012) and Fard et al. (2014), we did not observe an effect on antibody response to NDV and IBV vaccinations. Furthermore, we did not detect an effect on peripheral blood immune cell percentages (lymphocytes, heterophils, or monocytes). Nevertheless, we observed significantly elevated CD4+ T cells that are expected to be mainly Th cells. Th cells play a role in both cellular and humoral immune responses and are critical for B cell differentiation and antibody production (Sharma and Tizard, 1984). Their increased percentage in mushroom supplemented hens can be an indication of a beneficial effect on immune function of laying hens. Another immune and stress parameter that has been studied in the context of OMW supplementation is the ratio of peripheral blood H:L, and increased H:L is often used as an indicator of stress (Madej et al., 2024). Hassan et al. (2020) and Fard et al. (2014) reported that H:L ratio was significantly decreased in mushroom-supplemented broilers. In the present study, no effect on H:L ratio was observed, in agreement with Toghyani et al. (2012). Results of the present study on H:L ratio may be probably explained by the fact that hens were not exposed to any stressful conditions and therefore there was no chance for dietary OMW to express its potentially beneficial effect on reducing stress.

The fatty acid profile of egg yolks was favorably altered as a consequence of dietary supplementation of laying hens with OMW. A linear increase in PUFAs, and a relevant decrease in SFAs was detected with most notable effects observed for the highest inclusion rate of 4%. This fatty acid profile change is mainly due to the increase in linoleic acid (C18:2n-6) and the decrease in palmitic acid (C16:0). The same trend, for the same yolk FAs has been also reported by Hwang (2012), when hens were fed with shiitake mushrooms, and these authors attributed their results to the relevant FA profile of the feed provided to hens. However, the observed differences in FA profile in the present study cannot be attributed to corresponding, differences in the FA profile of the experimental diets. Neither linoleic acid (C18:2n-6) nor palmitic acid (C16:0) concentrations differed between control and experimental diets, since the small inclusion rates of oyster mushroom waste (1 to 4%) did not significantly affect the diets’ FA profile. Therefore, yolk FA profile differences may be attributed to the protective effect of natural antioxidants on yolk FA oxidation (Lioliopoulou et al., 2023). As mentioned previously, yolks from OMW fed hens displayed improved antioxidant capacity in the present study. Thus, the natural antioxidants present in OMW may have prevented FA oxidation and consequently positively affected egg PUFAs content, resulting in higher PUFA percentage in eggs from OMW fed hens. On the other hand, the fatty acid profile in animal products may be regulated by gut microflora, through the so-called “host-gut microbiota-metabolism axis” (Zhou et al., 2024) and the gut microflora of hens in the present study may have been altered by OMW feeding. However, because we haven't analyzed the gut microbiome, we cannot speculate that the gut microflora is responsible for the observed effects on FA profile. Future research on gut microbiome may further elucidate the mechanisms involved in yolk FA profile regulation when hens are fed with OMW.

In conclusion, dietary supplementation with *P. ostreatus* waste, at 1, 2 and 4 g per 100g of feed, improved the oxidative stability of both fresh and stored eggs, at room temperature for 28 d, or in the refrigerator for 90 d. Serum cholesterol content was reduced but this reduction was not observed in egg yolks. However, yolk SFAs were reduced and PUFAs were increased, with increased levels of OMW, resulting in an improved nutritional profile of eggs. Since no adverse effects were observed on performance and egg quality, apart from a slight decrease in intensity of yolk color, the OMW may be successfully employed in laying hens’ diets. Nevertheless, further research, in a larger scale and for a longer trial period, is required in order to elucidate nodes of action of OMW to laying hens. Utilization of an agro-industrial byproduct in poultry feed, that otherwise may pose a threat to the environment in the form of pollution, would effectively contribute to sustainability of both the poultry and mushroom production sectors. Expected benefits are not limited to the environment, but to consumers and farmers as well.

## DISCLOSURES

The authors declare the following financial interests/personal relationships which may be considered as potential competing interests: Ioannis Politis reports financial support was provided by European Regional Development Fund. If there are other authors, they declare that they have no known competing financial interests or personal relationships that could have appeared to influence the work reported in this paper.
